# The Study of Inebriety

**Published:** 1900-09-01

**Authors:** 


### EDITOR'S LETTER-BOX.

[Our Correspondents are reminded that prolixity is a great bar to publication, and that brevity of style and conciseness of statement greatly facilitate early insertion.]

### THE STUDY OF INEBRIETY.

Dr. Wm. Wynn Westcott, M.B., D.P.H., coroner, and president of the Society for the Study of Inebriety, writes to correct an error which has crept into a leading article in The Hospital for August 18th, where the committee appointed by the society to investigate the relation between heredity and inebriety is spoken of as a "committee of teetotallers." This, he says, is an error, as only a minority of the members can be so called.

